# The Longitudinal Association of Subclinical Hearing Loss With Cognition in the Health, Aging and Body Composition Study

**DOI:** 10.3389/fnagi.2021.789515

**Published:** 2022-03-01

**Authors:** Alexander Chern, Alexandria L. Irace, Rahul K. Sharma, Yuan Zhang, Qixuan Chen, Justin S. Golub

**Affiliations:** ^1^Department of Otolaryngology—Head and Neck Surgery, Columbia University Irving Medical Center, NewYork-Presbyterian Hospital, Columbia University Vagelos College of Physicians and Surgeons, New York, NY, United States; ^2^Department of Otolaryngology—Head and Neck Surgery, Weill Cornell Medical College and NewYork-Presbyterian Hospital, New York, NY, United States; ^3^Department of Biostatistics, Mailman School of Public Health, Columbia University, New York, NY, United States

**Keywords:** subclinical hearing loss, hearing loss, hearing aids, cognition, cognitive decline, dementia, cognitive impairment, quality of life

## Abstract

**Objectives:**

To examine the longitudinal association between subclinical hearing loss (SCHL) and neurocognitive performance.

**Design:**

Longitudinal analyses were conducted among 2,110 subjects who underwent audiometric testing in a US multi-centered epidemiologic cohort study. The primary exposure was better ear hearing (pure tone average). SCHL was defined as hearing ≤ 25 dB. The primary outcome was neurocognitive performance, measured by Digit Symbol Substitution Test (DSST), Modified Mini Mental State Examination (3MS), and CLOX1. Linear mixed models were performed to assess the longitudinal association between hearing and cognitive performance, adjusting for covariates. Models were fit among all individuals and among individuals with SCHL only.

**Results:**

Among 2,110 participants, mean (SD) age was 73.5 (2.9) years; 52.3% were women. Mean (SD) better ear pure tone average was 30.0 (13.1) dB. Mean follow-up was 9.1 years (range 3–16). Among all participants, worse hearing was associated with significantly steeper cognitive decline measured by the DSST [0.054-point/year steeper decrease per 10 dB worse hearing, 95% confidence interval (CI): 0.026–0.082] and 3MS (0.044-point/year steeper decrease per 10 dB worse hearing, CI: 0.026–0.062), but not CLOX1. Among those with SCHL, worse hearing was associated with significantly steeper cognitive performance decline as measured by DSST (0.121-point/year steeper decrease per 10 dB worse hearing, CI: 0.013–0.228), but not CLOX1 or 3MS.

**Conclusion:**

Among those with SCHL, worse hearing was associated with steeper cognitive performance declines over time as measured by DSST. The relationship between hearing loss and cognition may begin at earlier levels of hearing loss than previously recognized.

## Introduction

Age-related hearing loss (ARHL) is highly prevalent and notably undertreated in the elderly. Approximately two thirds of adults older than 70 years have hearing loss (HL) (Goman and Lin, 2016; Sharma et al., 2020), but fewer than 20% of adults affected by HL obtain treatment (e.g., hearing aids or cochlear implants) (Chien and Lin, 2012). As the global population grows and ages, the number of people with HL is increasing rapidly. For individuals aged 12 years and older in the United States, nearly 1 in 8 has bilateral HL (30 million or 12.7% of Americans). This estimate increases to nearly 1 in 5 (48.1 million or 20.3%) when including individuals with unilateral HL (Lin et al., 2011b). There is also a significant health burden that comes with untreated HL—an estimated annual global cost of US$750 billion (World Health Organization, 2021a).

Cognitive impairment, dementia, and depression are all highly prevalent and disabling disorders of later life. Recognition of ARHL as a potential risk factor for such neuropsychiatric conditions of older life is a new development that has not been previously prioritized in the management of patients with or at risk for such conditions. Recent prospective cohort studies have shown that ARHL confers an independent risk of age-related conditions such as cognitive impairment and incident dementia in subjects with normal cognition at baseline (Lin et al., 2011a,^2013^; Gallacher et al., 2012; Quaranta et al., 2015; Deal et al., 2017b; Golub et al., 2017, 2020b; Chern and Golub, 2019; Brewster et al., 2021a,b; Sharma et al., 2021). Moreover, the population attributable fracture (PAF) of HL for dementia (i.e., the percentage reduction in incident dementia if HL were completely eliminated) has been estimated at 8.2%. This PAF of HL is higher than the PAF of any other individual modifiable risk factor, including smoking (5.2%), depression (3.9%), social isolation (3.5%), hypertension (1.9%), and diabetes (1.1%) (Livingston et al., 2020). The high relative risk and prevalence of ARHL makes it a plausible target in preventative strategies for neuropsychiatric conditions of later life. ARHL is also severely undertreated, easily diagnosed, and treatable compared to non-modifiable risk factors such as age and family history (Chern and Golub, 2019). However, studies need to establish causality between HL and such disorders before making definitive recommendations regarding treatment of ARHL as a means to reduce cognitive decline and incident dementia.

The relationship between ARHL and neuropsychiatric conditions of older life appears to be dose-dependent. In other words, the risks of cognitive impairment, incident dementia, and depressive symptoms increase as the severity of HL increases (Lin et al., 2011a,^2013^; Deal et al., 2017b; Golub et al., 2017, 2019). Though studies have shown this phenomenon is first seen with mild HL (e.g., 26–40 dB), the association between cognition or mood and HL in the range of subclinical hearing loss [or “normal” hearing, i.e., a pure tone average (PTA) ≤ 25 dB] (World Health Organization, 2021b) has not been widely investigated. These data may inform the definition of HL and when HL treatment should begin, as there are currently no strong evidence-based guidelines. Our group has demonstrated a robust and independent association between subclinical hearing loss (SCHL, i.e., hearing ≤ 25 dB) and cognitive impairment (Golub et al., 2020a) in cross-sectional studies. To our knowledge, there is currently only one existing study examining the longitudinal association between SCHL and cognitive impairment (Irace et al., 2021). However, this study was limited by including primarily highly educated white individuals at a single geographic location. The objective of the present study is to examine the longitudinal association between SCHL and cognitive impairment using a large US multi-centered and multi-ethnic cohort study, where findings may better generalize to the US population.

## Materials and Methods

### Subjects

The Health, Aging and Body Composition (ABC) Study is a prospective cohort study of 3,075 community-dwelling black (42%) and white (58%) volunteers aged 70–79 years at baseline (in 1997–1998) sampled from Medicare enrollees living in Memphis, Tennessee or Pittsburgh, Pennsylvania (NIA, 2013). Eligibility criteria for Health ABC included no self-reported difficulty with mobility (i.e., able to walk a quarter mile and climb 10 steps without resting) or disability (i.e., difficulty performing activities of daily life), no life-threatening cancers, and no plans to leave the area within 3 years. Demographic information including age (years), race (white or black), sex, and education (less than high school, high school, or postsecondary) was collected at baseline. Self-reported smoking status (never, former, current), hearing aid use, presence of hypertension, presence of diabetes, and history of stroke were also reported.

**FIGURE 1:**
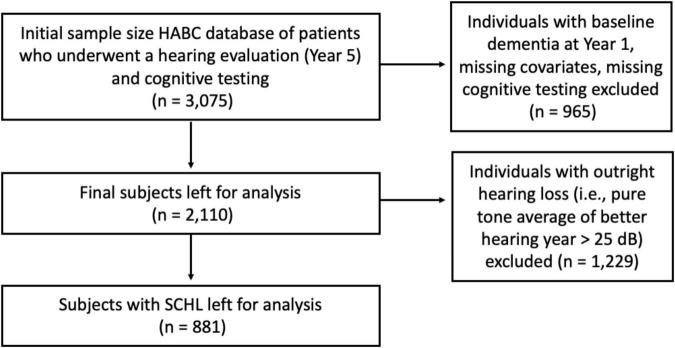
Application of inclusion and exclusion criteria on initial subjects.

The initial sample size was 3,075 subjects. Individuals who underwent a hearing evaluation (i.e., pure tone audiometry) and cognitive testing were included. Individuals with baseline dementia in 1997–1998 (Year 1) and missing data (e.g., covariates, cognitive testing) were excluded. The dementia criteria utilized was the same as previous Health ABC studies (Deal et al., 2017b). After applying inclusion and exclusion criteria, 2,110 subjects were left for analysis. See the flow diagram in Figure 1.

### Exposure: Hearing Loss

Hearing loss was measured using pure tone audiometry. Audiometry was conducted in 2001–2002 at Year 5 of the Health ABC study. A portable audiometer (Maico MA40) and supra-aural earphones (TDH 39) were used to obtain air conduction thresholds in each ear. Audiometric testing was performed in a sound booth that met ANSI standards, and hearing thresholds were measured in decibels (dB) hearing level. The pure-tone average (PTA) in the better hearing ear was calculated using hearing thresholds at 0.5, 1, 2, and 4 kHz. SCHL, a term espoused by our group, is defined as imperfect hearing that is classically defined as normal (Golub et al., 2020a,c). The widely used adult PTA cutoff of 25 dB was used to divide participants into SCHL (PTA ≤ 25 dB hearing level) and outright HL (PTA > 25 dB hearing level). The primary exposure variable was hearing as measured by the PTA of the better hearing ear, defined continuously.

### Outcome: Cognitive Performance

Cognitive impairment was assessed using widely used neurocognitive tests: the CLOX1, the 3MS examination, and the Digit Symbol Substitution Test (DSST) (Deal et al., 2017b). Per Health ABC study protocol, subjects underwent neurocognitive testing every 1–2 years from Years 1 to 16. All outcome variables were treated as continuous measures.

The CLOX1 is a validated clock-drawing test designed to measure executive impairment. Subjects are instructed to “draw me a clock that says 1:45; set the hands and numbers on the face so that a child could read them.” Scoring is based on whether certain organizational and sequential elements were achieved while drawing the clock (e.g., “Does figure resemble a clock?”; “12, 6, 3, and 9 placed first?”) (Royall et al., 1998).

The 3MS is a validated screening test for cognitive decline and dementia. It samples a broad variety of cognitive functions, including attention, concentration, orientation to time and place, long-term and short-term memory, language, constructional praxis, abstract thinking, and list-generating fluency (Teng and Chui, 1987).

The DSST is a validated cognitive test that requires participants to fill in a series of symbols correctly coded within 90 s (Wechler, 1981). It assesses working memory, motor speed, attention, and visuo-perceptual functions [scanning and ability to write/draw (i.e., basic manual dexterity); Wechler, 1981; Jaeger, 2018].

### Covariates

Demographic information and comorbidities were collected at Year 1 (1997–1998). Those relevant were included as covariates that may confound the association between HL and cognitive decline in our multivariable regression models. Demographics included age (years), race (white or black), sex (male or female), and education (less than high school, high school, or postsecondary education). Smoking was assessed with self-report (never, current, or former). Diabetes, stroke, and hypertension were considered present if the subject self-reported a prior physician diagnosis. Hearing aid use was self-reported at baseline in Year 5.

### Statistical Analysis

The primary analyses were performed as follows. Generalized longitudinal mixed effect models were used to model the association of PTA of the better hearing ear (defined continuously, measured year 5) with cognitive measures (CLOX1, 3MS, DSST) over time from Years 1 to 16. Both univariable and multivariable models (adjusting for covariates that may be associated with both the exposure and the outcomes) were performed. Interaction between hearing and year was included in the models to allow the association between hearing and cognitive performance to vary across time. Prior to the analyses, subjects with baseline dementia and missing variables at Year 1 were excluded; the outcomes were investigated for outliers, then transformed to either achieve normally distributed outcomes/residuals or modeled via an appropriate link function. Covariates were investigated similarly and transformed (if necessary), to minimize influential effect of outliers at baseline. Models were fit first among all subjects and then among subjects with SCHL only. Statistical analyses were performed in R (R Foundation for Statistical Computing) using RStudio SAS 9.4 (Cary, NC), and Stata (College Station, TX) (SAS Institute, 2013; RStudio Team, 2015; StataCorp, 2019).

Sensitivity analyses were performed in similar fashion to the above, except from Years 5 to 16 only. This was done because our primary analysis included timepoints prior to Year 5 during which time the exposure (HL) was measured.

The Columbia University Irving Medical Center Institutional Review Board provided a Not Human Subjects Research determination (Under 45 CFR 46) for this study.

## Results

Table 1 demonstrates baseline subject demographic characteristics. During the study period (1997–2013; Years 1–16), 2,110 subjects were dementia-free and had audiometric testing in Year 5. Of these participants, 881 had SCHL (i.e., pure tone average in better ear of ≤ 25 dB hearing level). The mean age of all subjects was 73.5 years; 52.3% were women. Mean pure tone average of the better hearing ear was 30.1 dB (*SD* = 13.1 dB). Mean follow-up of subjects was 9.1 (range 3–16) years.

**TABLE 1:** Mean (SD) and frequency (%) of demographic variables and comorbidities among all subjects and individuals with SCHL.

Characteristic	All (*n* = 2,110)	With SCHL (*n* = 881)
Age, in years, mean (SD), IQR	73.5 (2.9), 5	72.8 (2.7), 4
Race, black, n (%)	788 (37.4%)	408 (46.3%)
Sex, female, n (%)	1,105 (52.4%)	543 (61.6%)
Education level, n (%)		
Less than high school	460 (21.8%)	183 (20.8%)
High school	696 (33.0%)	290 (32.9%)
Postsecondary education level	954 (45.2%)	408 (46.3%)
Pure tone average of the better hearing ear, mean (SD), IQR	30.1 (13.1), 18.8	18.2 (5.0), 7.5
Smoking status, n (%)		
Never	975 (46.2%)	449 (51.0%)
Current smoker	172 (8.2%)	75 (8.5%)
Former smoker	963 (45.6%)	357 (40.5%)
Diabetes, n (%)	285 (13.5%)	113 (12.8%)
History of stroke, n (%)	43 (2.0%)	13 (1.5%)
Hypertension, n (%)	1,044 (49.5%)	450 (51.1%)
Hearing aid use, n (%)	176 (8.3%)	4 (0.5%)

Table 2 shows results from the univariable (unadjusted) linear mixed effects models estimating the associations of HL in the better hearing ear with cognitive decline among all subjects. Table 3 shows results from the multivariable linear mixed effects models of the same. Among all participants, worse hearing was associated with a significantly steeper decline in cognitive performance over time as measured by the DSST, adjusting for covariates (estimate –0.054 for every 10 dB worse hearing, 95% confidence interval –0.082 to –0.026, *p* < 0.01). In other words, a 10-dB worsening in hearing was associated with a 0.054-point steeper decline per year. Worse hearing was also associated with a significantly steeper decline over time in all participants as measured by the 3MS (–0.044, –0.062 to –0.025 per 10 dB, *p* < 0.01), adjusting for covariates. In other words, a 10-dB worsening in hearing was associated with a 0.044-point steeper decline per year. Worse hearing was not significantly associated with a steeper decline in cognitive performance as measured by the CLOX1 test, adjusting for covariates. Sensitivity analyses performed from Years 5 to 16 (Supplementary Tables 1, 2) also demonstrated a significantly steeper decline over time in all participants as measured by the 3MS and DSST, but not the CLOX1.

**TABLE 2:** Univariable (unadjusted) linear mixed models.

Cognitive test	Estimate (95% confidence interval)	*p*-value
DSST	–0.054 (–0.082 to –0.026)	< 0.001*
3MS	–0.044 (–0.062 to –0.025)	< 0.001*
CLOX1	0.002 (–0.010 to 0.013)	0.786

Longitudinal association between 10-dB worsening in better ear hearing and cognitive decline in all subjects (n = 2,110). *Indicates p < 0.05. DSST, Digit Symbol Substitution Test; 3MS, Modified Mini-Mental State Exam.

**TABLE 3:** Multivariable linear mixed models.

Cognitive test	Estimate (95% confidence interval)	*p*-value
DSST	–0.054 (–0.082 to –0.026)	< 0.001*
3MS	–0.044 (–0.062 to –0.026)	< 0.001*
CLOX1	0.001 (–0.010 to 0.012)	0.842

Longitudinal association between 10-dB worsening in better ear hearing and cognitive decline in all subjects (n = 2,110), adjusting for age, race, sex, education level, smoking status, diabetes, history of stroke, hypertension, hearing aid use. *Indicates p < 0.05. DSST, Digit Symbol Substitution Test; 3MS, Modified Mini-Mental State Exam.

Table 4 shows results from the univariable (unadjusted) linear mixed effects models estimating the associations of hearing loss in the better hearing ear with cognitive decline among subjects with SCHL. Table 5 shows results from the multivariable linear mixed effects models estimating the associations of hearing loss in the better hearing ear with cognitive decline among subjects with SCHL. Among participants with SCHL, worse hearing was associated with a significantly steeper decline in cognitive performance over time as measured by the DSST (–0.120, –0.227 to –0.012 per 10 dB, *p* = 0.029), adjusting for covariates. In other words, a 10-dB worsening in hearing was associated with a 0.120-point steeper decline per year. Worse hearing was not significantly associated with a steeper decline in cognitive performance as measured by the CLOX1 or 3MS, adjusting for covariates. However, the *p*-value for the 3MS (–0.064, –0.131 to 0.003 per 10 dB, *p* = 0.060) did approach significance. Sensitivity analyses performed from Years 5 to 16 (Supplementary Tables 3, 4) did not demonstrate a significantly steeper decline over time in all participants for any cognitive measures. Of note, power was reduced for the sensitivity analysis (3,694 observations for the sensitivity analysis vs. 4,569 observations for the primary analysis in a sample size of 881 subjects).

**TABLE 4:** Univariable (unadjusted) linear mixed models.

Cognitive test	Estimate (95% confidence interval)	*p*-value
DSST	–0.125 (–0.233 to –0.017)	0.023*
3MS	–0.065 (–0.132 to 0.002)	0.059
CLOX1	–0.002 (–0.042 to 0.039)	0.935

Longitudinal association between 10-dB worsening in better ear hearing and cognitive decline in only subjects with SCHL. *Indicates p < 0.05. DSST, Digit Symbol Substitution Test; 3MS, Modified Mini-Mental State Exam.

**TABLE 5:** Multivariable linear mixed models.

Cognitive test	Estimate (95% confidence interval)	*p*-value
DSST	–0.120 (–0.227 to –0.012)	0.029*
3MS	–0.064 (–0.131 to 0.003)	0.060
CLOX1	0.000 (–0.041 to 0.041)	0.999

Longitudinal association between 10-dB worsening in better ear hearing and cognitive decline in only subjects with SCHL (n = 881), adjusting for age, race, sex, education level, smoking status, diabetes, history of stroke, hypertension, hearing aid use. * Indicates p < 0.05. DSST, Digit Symbol Substitution Test; 3MS, Modified Mini-Mental State Exam.

## Discussion

Prior studies have shown an independent association between hearing loss (HL) and cognitive decline/dementia (Lin et al., 2011a,^2013^; Gallacher et al., 2012; Quaranta et al., 2015; Deal et al., 2017b; Golub et al., 2017; Chern et al., 2021). Recent cross-sectional data has shown that this relationship persists even within the range of “normal hearing” (i.e., PTA ≤ 25 dB HL) also known as SCHL (Golub et al., 2020a). Our novel study employed a US multi-centered, multi-ethnic epidemiologic cohort to examine this association longitudinally. Among all subjects, worse hearing was associated with a significantly steeper decline in cognitive performance as measured by the DSST and 3MS over time. Among subjects with SCHL, worse hearing was associated with steeper declines in cognitive performance as measured by the DSST over time.

Our findings demonstrate a longitudinal relationship between SCHL and cognitive decline on a test of speed and attention. This further supports prior cross-sectional studies and hints at the possibility of a causal mechanism between HL and cognition that begins while HL is still within the normal range of hearing, or SCHL. Moreover, these results establish a temporality and directionality to the previously established association between SCHL and cognition, as the exposure (SCHL) was generally present before the outcome (cognitive decline). We additionally confirm findings of a prior recent study by our group (Irace et al., 2021) and extend it to a multi-centered and multi-ethnic population.

Although a definitive causal mechanism has yet to be established, there are several plausible mechanistic pathways explaining this hearing-cognition relationship (Chern and Golub, 2019). One potential pathway is that ARHL confers a greater risk of social isolation in older adults (Weinstein and Ventry, 1982; Strawbridge et al., 2000; Mick et al., 2014), which in turn increases their risk of worse cognition. Indeed, social interaction, emotional and intellectual stimulation have been shown to be protective against cognitive decline (Fratiglioni et al., 2000). Another mechanism is that ARHL may cause detectable changes in brain structure, which then increases the risk of cognitive decline and dementia. Impoverished auditory signals and decreased cortical stimulation may affect neural networks and brain structure. For example, studies have shown that older adults with HL have decreased volumes in regions responsible for auditory processing (Peelle et al., 2011; Eckert et al., 2012), as well as the entire brain (Driscoll et al., 2009; Lin et al., 2014), which may also have downstream effects on cognitive processes also dependent on these same regions. A final potential mechanism is increased cognitive load—studies have shown that under difficult auditory environments, individuals with HL may be burdened with a greater cognitive load and may readily exhaust their cognitive resources compared to normal-hearing peers. Increased effort with auditory processing can occur at the expense of other cognitive processes, such as working memory and learning (Pichora-Fuller and Singh, 2006; Tun et al., 2009). Individual differences in ability to optimize cognitive performance through differential recruitment of brain networks may allow some individuals to better cope with neuropathology (e.g., Alzheimer’s disease or traumatic brain injury) than others (Neuropathology Group. Medical Research Council Cognitive Function and Aging Study, 2001; Savva et al., 2009; Stern, 2012). It is also possible that a common (i.e., non-causal, or confounding) mechanism exists, which causes the development of both HL and cognitive decline. Examples of this include microvascular disease or some other unknown common neuropathologic processes (Chern and Golub, 2019). In this study, we adjusted for multiple potential confounders in our models.

While a significant longitudinal association was found between SCHL and cognitive decline as measured by DSST scores over time, this was not observed for other measures of cognition (3MS and CLOX1). Several explanations may justify these findings. The DSST measures a variety of cognitive functions, including motor speed, attention, visuo-perceptual processing, and working memory. Our findings appear to align with previous research suggesting that the DSST is sensitive to mild cognitive changes in individuals with relatively good cognitive function; lower DSST scores have shown to be associated with increased risk of developing subclinical or clinical cognitive disorders in individuals with normal baseline cognition (Rosano et al., 2016). The longitudinal association between SCHL and the 3MS approached significance; the smaller sample size of the SCHL group (*n* = 883) compared to all subjects (*n* = 2,110) may have been less powered to detect this effect. Although the CLOX1 test is designed to assess executive function through a clock-drawing exercise, individuals with subclinical or mild cognitive impairment may draw a clock from long-term memory (rather than as a series of steps as intended by the assessment) and only demonstrate minor difficulty with drawing the numbers (with adequate spacing) and clock hands. Indeed, mild cognitive impairment tends to affect short-term (episodic) memory first, rather than long-term memory (Levin, 2021); the CLOX1 may not have appropriately captured the level of cognitive impairment in subjects with SCHL, who may only have just begun to exhibit signs of mild cognitive impairment. As SCHL progresses to outright HL (i.e., PTA > 25), these subjects would be at increased risk for developing clinically detectable cognitive impairment. A final possibility that may explain our results is that there is no longitudinal association between SCHL and cognitive decline; however, this is unlikely given the previous longitudinal studies that have suggested a dose-dependent relationship between HL and cognition, recent cross-sectional work that have demonstrated an independent association between SCHL and cognition, and the plausible causal mechanisms explaining this association (Chern and Golub, 2019; Golub et al., 2020a).

Because hearing, the exposure, was not measured until year 5, we also performed sensitivity analyses restricted to years 5 through 16 only. Results were similar across all participants, but significance was lost among those with SCHL. The negative finding among those with SCHL may be due to lower power from the reduced observations from omitting years 1 through 4. Including the outcomes for 4 years prior to measurement of hearing in year 5 is reasonable because age-related HL progresses slowly at under 1 dB/year even in later life (Sharma et al., 2020). Moreover, other published studies using the Health ABC have also included outcomes from Year 1 and Year 5 in their primary analyses (Chen et al., 2015; Kamil et al., 2016; Deal et al., 2017b).

Although a causal relationship between HL and cognition has not yet been definitively established, there is low risk of widely testing for and treating HL. While direct treatment of SCHL itself (i.e., with amplification) seems excessive when most adults with moderate-or-worse HL do not get treatment, there are several strategies that can be employed to mitigate the potential adverse effects of SCHL. Optimizing the acoustic environment through use of microphones and speakers in public spaces is one such treatment. Other strategies include improving visual cues (e.g., lip reading) with bright lighting, minimizing background noise, creating seating arrangements to optimize verbal communication, preferential seating for individuals who struggle to hear, and reducing reverberation (i.e., with sound-absorbing materials). Indeed, the effects of mask-wearing and virtual meetings during the recent COVID-19 pandemic has highlighted how suboptimal auditory environments can disrupt communication and cause unforeseen effects in both individuals with hearing loss and their normal-hearing peers (Ribeiro et al., 2020; Charney et al., 2021; Wilson et al., 2021). Masks can degrade the speech signal and attenuate sound levels anywhere from 3 to 4 dB for typical masks and up to 12 dB for N-95 masks (Goldin et al., 2020); an individual with “perfect” hearing who listens to speech transmitted from behind a mask would essentially be listening under conditions of SCHL. Even individuals with baseline normal hearing are known to experience increased listening effort from difficulties interpreting non-verbal cues in a poor auditory environment (i.e., decreased audio quality and audiovisual desynchrony) in certain virtual meeting settings; previous studies have shown that listening is particularly effortful in demanding auditory conditions, such as a noisy background or when the listeners themselves have auditory processing deficits (e.g., SCHL or outright HL) (Sklar, 2020). Some studies supporting this “information degradation hypothesis” suggest that there are short-term and possibly long-term effects of experiencing such conditions on cognitive performance (Pichora-Fuller and Singh, 2006). These examples highlight the importance of optimizing the auditory environment and facilitating communication to prevent negative downstream cognitive effects in both individuals with HL and individuals with what is classically considered “normal hearing.”

Our study has several limitations. SCHL progresses to outright HL over time. Despite including only subjects with baseline SCHL at Year 5 of the study, some participants may have progressed to outright HL during the follow-up period (i.e., from Years 6 to 16). The development of outright HL (rather than SCHL) may have conferred the risk for cognitive decline in our analysis. Practice effects (i.e., improvements in cognitive test performance due to the ability of a subject to learn and adjust with repeated exposure to the test materials) were not accounted for in our study. However, the frequency of repeated cognitive testing was low (i.e., at least 1–2 years between repeated tests); studies have suggested a performance plateau upon low-frequency testing (Bartels et al., 2010). While our study attempted to control for confounding variables in our multivariable analysis, it is not possible to completely control for confounding in an observational study, since not all confounders are known or measurable. As previously stated, our study was also somewhat limited by our sample size—the longitudinal association between SCHL and cognitive performance as measured by the 3MS approached significance. The Health ABC cohort is biracial and only includes white and black subjects; this may limit the external validity of findings. Lastly, although the temporal relationship established by our findings suggests the possibility of a causal mechanism between HL and cognition that begins while HL is still subclinical and within the range of normal hearing, we cannot definitively establish a causal relationship without a randomized controlled trial.

Future directions include conducting randomized controlled trials to examine a causal relationship between HL and cognitive decline and thus the utility of targeting HL as a modifiable risk factor for cognitive decline and dementia. This is already underway with the Aging and Cognitive Health Evaluation in Elders (ACHIEVE) trial, which is the first randomized controlled trial aimed at determining the effectiveness of a best practices hearing intervention (i.e., hearing aid) compared to a successful aging intervention on reducing cognitive decline and preventing dementia (Deal et al., 2017a; Sanchez et al., 2020). Employing other measures of HL aside from pure tone audiometry may provide more sensitivity or ecological validity. Another potential future direction is to revisit the cutoff of what constitutes adult HL. The commonly used 25 dB threshold for defining adult HL is arbitrary (Gatlin and Dhar, 2021). The WHO now recommends 20 dB (World Health Organization, 2021a). In 2008, the Global Burden of Disease Expert Group on Hearing Impairment recommended this change based on clinical experience of this expert group and existing literature suggesting the normal 25 dB hearing level threshold was not in agreement with the functional experience of persons with what has been called “mild” or “slight” hearing impairment in the literature (i.e., ≤ 25 dB hearing level)—these individuals may also experience hearing problems (Olusanya et al., 2019). Further elucidating the relationship between SCHL and other conditions of aging will provide evidence of whether this threshold is too lax, especially if there are established clinical consequences of SCHL (i.e., increased risk of cognitive decline and dementia).

In summary, a longitudinal association was established between SCHL and cognitive decline as measured by the DSST. Our findings suggest that it is possible that changes in cognitive performance may occur before the traditional threshold of HL is reached. Further studies are necessary to determine exactly when in the spectrum of HL this observable relationship begins and the utility of targeting HL as a modifiable risk factor for cognitive decline and dementia.

## Data Availability Statement

Publicly available datasets were analyzed in this study. This data can be found here: https://healthabc.nia.nih.gov/.

## Author Contributions

AC and JG contributed to the conception, design, data interpretation, and edited the final manuscript for submission. AI and RS developed the initial refinement of the dataset. YZ and QC contributed to the data analysis and interpretation of the dataset. All authors were involved in writing the first draft of the manuscript, read, and approved the submitted version.

## Conflict of Interest

JG travel expenses for industry-sponsored meetings (Cochlear, Advanced Bionics, Oticon Medical), consulting fees or honoraria (Oticon Medical, Auditory Insight, Optinose, Abbott, Decibel Therapeutics), department received unrestricted educational grants (Storz, Stryker, Acclarent, 3NT, Decibel Therapeutics). The remaining authors declare that the research was conducted in the absence of any commercial or financial relationships that could be construed as a potential conflict of interest.

## Publisher’s Note

All claims expressed in this article are solely those of the authors and do not necessarily represent those of their affiliated organizations, or those of the publisher, the editors and the reviewers. Any product that may be evaluated in this article, or claim that may be made by its manufacturer, is not guaranteed or endorsed by the publisher.
