# Supplementary material for: The Longitudinal Association of Subclinical Hearing Loss With Cognition in the Health, Aging and Body Composition Study
Source: Front Aging Neurosci. 2022 Mar 1;13:789515. doi: 10.3389/fnagi.2021.789515 (PMC8923153; doi:10.3389/fnagi.2021.789515)
Supplement: Supplementary file 1 [file Table_1.pdf]

Supplementary Table 1. Sensitivity analysis (starting at Year 5). Univariable (unadjusted) linear mixed models. Longitudinal association between 10-dB worsening in better ear hearing and cognitive decline in all subjects (n=2,110). * indicates p < 0.05. DSST = Digit Symbol Substitution Test. 3MS = Modified Mini-Mental State Exam.

Cognitive Test[‡]	Estimate (95% Confidence Interval)	p-value
DSST	-0.062 (-0.107 to -0.018)	0.006*
3MS	-0.032 (-0.062 to -0.001)	0.042*
CLOX1	0.008 (-0.008 to 0.024)	0.334

Supplementary Table 2. Sensitivity analysis (starting at Year 5). Multivariable linear mixed models. Longitudinal association between 10-dB worsening in better ear hearing and cognitive decline in all subjects (n=2,110), adjusting for age, race, sex, education level, smoking status, diabetes, history of stroke, hypertension, hearing aid use. * indicates p < 0.05. DSST = Digit Symbol Substitution Test. 3MS = Modified Mini-Mental State Exam.

Cognitive Test[‡]	Estimate (95% Confidence Interval)	p-value
DSST	-0.062 (-0.106 to -0.017)	0.006*
3MS	-0.032 (-0.063 to -0.002)	0.037*
CLOX1	0.007 (-0.009 to 0.023)	0.368

Supplementary Table 3. Sensitivity analysis (starting at Year 5). Univariable (unadjusted) linear mixed models. Longitudinal association between 10-dB worsening in better ear hearing and cognitive decline in only subjects with SCHL. * indicates $p < 0.05$. DSST = Digit Symbol Substitution Test. 3MS = Modified Mini-Mental State Exam.

Cognitive Test	Estimate (95% Confidence Interval)	p-value
DSST	-0.087 (-0.255 to 0.080)	0.305
3MS	-0.059 (-0.167 to 0.020)	0.291
CLOX1	0.038 (-0.021 to 0.097)	0.204

Supplementary Table 4. Sensitivity analysis (starting at Year 5). Multivariable linear mixed models. Longitudinal association between 10-dB worsening in better ear hearing and cognitive decline in only subjects with SCHL (n=881), adjusting for age, race, sex, education level, smoking status, diabetes, history of stroke, hypertension, hearing aid use. * indicates $p < 0.05$. DSST = Digit Symbol Substitution Test. 3MS = Modified Mini-Mental State Exam.

Cognitive Test	Estimate (95% Confidence Interval)	p-value
DSST	-0.076 (-0.242 to 0.091)	0.373
3MS	-0.060 (-0.168 to 0.049)	0.281
CLOX1	0.039 (-0.019 to 0.097)	0.188
